# Physiological and photosynthetic characteristics of indica Hang2 expressing the sugarcane *PEPC* gene

**DOI:** 10.1007/s11033-014-3070-4

**Published:** 2014-01-29

**Authors:** Ling Lian, Xiaowei Wang, Yongsheng Zhu, Wei He, Qiuhua Cai, Huaan Xie, Muqing Zhang, Jianfu Zhang

**Affiliations:** 1 Rice Research Institute, Fujian Academy of Agricultural Sciences, Fuzhou, Fujian Province 350018 People’s Republic of China; 2 Incubator of National key Laboratory of Fujian Crop Germplasm Innovation and Molecular Breeding between Fujian and Ministry of Sciences and Technology/Key Laboratory of Germplasm Innovation and Molecular Breeding of Hybrid Rice for South China, Ministry of Agriculture, Fuzhou, Fujian province 350003 People’s Republic of China; 3 South-China Base of National Key Laboratory of Hybrid Rice of China, Fuzhou, Fujian province 350003 People’s Republic of China; 4 National Engineering Laboratory of Rice, Fuzhou, Fujian province 350003 People’s Republic of China; 5 Sugarcane Research Institute, Fujian Agriculture and Forestry University, Fuzhou, Fujian province 350002 People’s Republic of China

**Keywords:** Phosphoenolpyruvate carboxylase gene (*PEPC* gene), C4 photosynthesis, Gene expression, Transgenic rice

## Abstract

Phosphoenolpyruvate carboxylase (PEPC) is known to play a key role in the initial fixation of CO_2_ in C4 photosynthesis. The *PEPC* gene from sugarcane (a C4 plant) was introduced into indica rice (Hang2), a process mediated by *Agrobacterium tumefaciens*. Integration patterns and copy numbers of the gene was confirmed by DNA blot analysis. RT-PCR and western blotting results showed that the *PEPC* gene was expressed at both the mRNA and protein levels in the transgenic lines. Real-time PCR results indicated that expression of the sugarcane *PEPC* gene occurred mostly in green tissues and changed under high temperature and drought stress. All transgenic lines showed higher PEPC enzyme activities compared to the untransformed controls, with the highest activity (11.1 times higher than the controls) being observed in the transgenic line, T34. The transgenic lines also exhibited higher photosynthetic rates. The highest photosynthetic rate was observed in the transgenic line, T54 (22.3 μmol m^−2^ s^−1^; 24.6 % higher than that in non-transgenic plants) under high-temperature conditions. Furthermore, the filled grain and total grain numbers for transgenic lines were higher than those for non-transgenic plants, but the grain filling (%) and 1,000-grain weights of all transgenic lines remained unchanged. We concluded that over-expression of the *PEPC* gene from sugarcane in indica rice (Hang2) resulted in higher PEPC enzyme activities and higher photosynthesis rates under high-temperature conditions.

**Electronic supplementary material:**

The online version of this article (doi:10.1007/s11033-014-3070-4) contains supplementary material, which is available to authorized users.

## Introduction

Photosynthesis is one of the major factors that affect rice yield. In total, 90 % of the crop yield directly comes from organic compounds created by photosynthesis. The yield per unit can increase significantly if the photosynthetic rate of the crop is increased to its full capacity [1–5]. The C4 photosynthesis pathway was discovered in the 1960s [6]. Since then, a number of studies have indicated that the C4 photosynthetic system is superior to the C3 system [7]. This photosynthetic superiority may result from increased CO_2_ concentration around Rubisco and the repression of photorespiration may increase the net photosynthetic rate. In addition, the efficiency of C4 Rubisco is higher than C3 catalysts. The C4 photosynthetic pathway also has an efficient photosynthetic transformation capacity. Therefore, the transformation of some C4 photosynthetic advantages, such as repressed photorespiration, increased utilization rates of water and nitrogen and improved photosynthetic rates, etc., into C3 crops may result in several plant improvements [8–10].

Many genes related to C4 photosynthesis (e.g., *NAD*-*ME*, *PEPC*, *PPDK* and *NADP*-*MDH*) have been successfully transformed into C3 genomes [11, 12]. The *NADP*-*ME* gene from sorghum was introduced into Nongken 58, a rice variety, using an *Agrobacterium*-mediated system, resulting in 1–7-fold elevation of NADP-ME activity and increased photoinhibition under high light intensity [13]. The *PEPC* gene from *Zea mays* was recently cloned and transformed into the rice genome using *Agrobacterium tumefaciens* [14]. This resulted in an increase in the density and area of stoma in the leaves and in the accumulation of dry matter in the transgenic rice. Oat (*Avena sativa*; C3) × maize (*Zea mays*; C4) addition lines (OMAs) allow the effects of individual maize chromosomes to be investigated in oat. When genes encoding phosphoenolpyruvate carboxylase (PEPC), orthophosphate dikinase, pyruvate and the 29-oxoglutarate/malate transporter in maize were expressed in oat, larger bundle sheath cells with increased cell wall lipid deposition were observed in oat leaves [15].

Phosphoenolpyruvate carboxylase, a key enzyme in photosynthesis, is an important carboxylase for plant cell metabolism. PEPC, which appears to fix CO_2_ in mesophyll cells to form C4 dicarboxylic acids, is more specific for inorganic carbon and improves the photosynthetic rate of C4 plants. The C4-type *PEPC* gene from sugarcane was transformed into a high-quality indica restorer line, “Hang2”, using *A. tumefaciens*, which has been extensively used to create super hybrid rice combinations in China. Here, we describe the expression pattern of the *PEPC* gene, *PEPC* gene expression under stress treatment in transgenic rice, PEPC enzyme activity and total nitrogen content in the transgenic plants; In addition, we report the physiological photosynthetic characteristics and yield characteristics of the transgenic rice.

## Materials and methods

### Transformation of indica rice (Hang2)

The plasmid pc1380, which contained the intact sugarcane *PEPC* gene driven by its endogenous promoter and *hph* as a marker, was used for the transformation via *A. tumefaciens*. The embryogenic calli of mature embryos of the rice cultivar indica restorer line, Hang2, were obtained and transformed using *A. tumefaciens*, as described by Datta [16]. The embryogenic calli were cultured in NB medium [17], containing 50 mg L^−1^ hygromycin, for three to four selection cycles with a 14-day duration per cycle. Embryogenic calli were selected for regeneration and the resulting plantlets were first grown in the culture solution before being transferred to pots. Putative primary transformants and subsequent seed progenies were grown in the transgenic network house.

### Polymerase chain reaction (PCR) assay and southern blot analysis

Genomic DNA was isolated from 1 month old plants by CTAB, and 25–50 ng of the DNA template was used for PCR analysis using *PEPC* gene specific primers (PEPC 1F and PEPC 1R). The reaction mixture contained 1 μL 10× buffer, 0.8 μL dNTP (2.5 mmol L^−1^), 0.4 μL PEPC 1F (10 μmol L^−1^), 0.4 μL PEPC 1R (10 μmol L^−1^), 0.1 μL *rTaq*, 25–30 ng DNA and enough water to bring the final volume to 10 μL. The PCR program was as follows: 94 °C denaturation for 5 min, 36 cycles of 94 °C for 30 s, 60 °C for 30 s and 72 °C for 1 min, extension for 10 min at 72 °C and final holding at 4 °C.

The plant genomic DNA was extracted from freshly harvested leaves of transgenic and non-transgenic control plants for southern blot analysis, following a modified CTAB method [18]. A total of 100 μg of DNA per sample was digested with *Hin*dIII at 37 °C overnight. The digested DNA was fractionated in 1 % (w/v) TAE agarose gels and then transferred to a Hybond-N nylon membrane (Amersham, Arlington Heights, IL, USA), according to the manufacturer’s instructions. The *Hygromycin* coding sequence was isolated from the plasmid and labeled using an AlkPhos direct labeling kit (Amersham, Arlington Heights, IL, USA) in order to make the hybridization probes.

### Total RNA isolation and reverse transcription polymerase chain reaction (RT-PCR) analysis of the transgenic rice

The total RNA was isolated from mature green leaves of the transgenic and control plants using Trizol reagent (Invitrogen, USA). RT-PCR was performed on 2 μg of the total RNA using specific primers for *pepc* (PEPC 2F and PEPC 2R) and *actin* (Actin 1F and Actin 1R), following a previously described method [19] (Table 1). The RT-PCR products were resolved on 1 % TAE-agarose gel.

**Table 1:** Primers used in this study

Primer name	Primer sequence (5′ → 3′)
PEPC 1F	ATCAAGGAGAAACTGGATG
PEPC 1R	TCAGGAAAGAACTAGACTGC
PEPC 2F	AGTCGGACATCGAGGAGACG
PEPC 2R	ACCACCCATCCAAGAACAGA
Actin 1F	CAT CTT GGC ATC TCT CAG CAC
Actin 1R	AAC TTT GTC CAC GCT AAT GAA
PEPC 3F	CCATCTGCTGGCTTCTGGAGTTTC
PEPC 3R	TTGTCGCCGCAGTCACACAGTG
Actin 2F	AGTGTCTGGATTGGAGGAT
Actin 2R	TCTTGGCTTAGCATTCTTG

### Quantitative reverse transcription PCR (qRT-PCR) analysis of *PEPC* gene expression

To analyze *PEPC* gene expression in different tissues of transgenic lines, green leaves, stems and roots of 3 weeks old transgenic seedlings were harvested. Expression of the *PEPC* gene under abiotic stress was also analyzed. The transgenic seedlings were placed onto filter papers to induce drought stress. Samples were taken after 0, 2, 4, 8, 12 and 24 h treatment. The transgenic seedlings were then placed in a 42 °C chamber and sampling took place after 0, 1, 3, 6, 12, and 24 h of treatment.

Total RNA was isolated from the samples using Trizol (Invitrogen, USA) and reverse transcription was performed on 2 μg of the total RNA using the RevertAid™ First Strand cDNA Synthesis Kit (Fermentas, LTU). The qRT-PCR was performed on a 7500 real-time PCR system (Applied Biosystems) with the Faststart Universal SYBR Green Master system (Roche, USA), following the manufacturer’s protocol. PEPC 3F and PEPC 3R were used as primers. Transcripts of the *Rice actin2* gene were amplified using Actin 2F and Actin 2R primers and used as the endogenous control. The relative quantitative method (△△CT) was used to evaluate the quantitative variation in the examined replicates.

### Extraction of resolved protein, western blotting and PEPC enzyme activity analysis

Green leaf segments, of about 5 cm in length, were harvested from the midsection of the uppermost fully expanded leaf and immediately frozen in liquid nitrogen until needed. Unless stated otherwise, the leaf samples were harvested at 11:00 a.m. on sunny days. To extract soluble proteins from the leaf segment, the samples were ground in extraction buffer containing 50 mmol L^−1^ Tris–HCl (pH 7.5), 1 mmol L^−1^ MgCl_2_, 5 mmol L^−1^ DDT, 1 mmol L^−1^ EDTA and 5 % (w/v) glycerol with a small amount of sea sand (Shanghai Sangon Biotech Co., Ltd., P.R. China). The homogenate was centrifuged at 13,000×*g* for 10 min at 4 °C and the resulting supernatant was collected as soluble protein extract. Protein concentration was determined using the method described by [20]. Bovine serum albumin was used as the standard. The precipitated samples were separated by SDS-PAGE and subjected to immune blot analysis using the PEPC antibody (antibodies for research, Agrisera). The assay mixture contained 50 mmol L^−1^ HEPES–KOH (pH 8.0), 5 mmol L^−1^ MgCl_2_, 2 mmol L^−1^ PEP, 0.2 mmol L^−1^ nicotinamide-adenine dinucleotide, 10 mmol L^−1^ NaHCO_3_ and 1.5 U of malate dehydrogenase (from pig heart; Roche Diagnostics, Basel, Switzerland) and the reaction was started by adding 20 μL of the enzyme extract.

### Measurement of photosynthetic characteristics

The flag leaf CO_2_ assimilation rate, stomatal conductance and internal CO_2_ concentration (Ci) were measured using a Li-6400 portable photosynthesis system (Li-Cor Inc., Lincoln, NE) and the chlorophyll relative content was measured using a SPAD-502 chlorophyll meter (Beijing Hezhong Bopu Technology Develop Co., Ltd.) between 10:00 a.m. and 12:00 a.m. under saturated photosynthetic photon flux density conditions (1,200 μmol m^−2^ s^−1^). The air temperature (T-air) in the greenhouse, as measured by the LI-6400 system, was 30 °C on average. Three fully expended flag leaves from each transgenic line and from the control plants were selected and measured at the beginning of the heading stage. The flow rate was maintained at 200–250 μmol s^−1^ so that the relative humidity inside the chamber was similar to ambient conditions. The measurements were made for 20 s immediately after a stable decrease in CO_2_ concentration was achieved inside the chamber. Statistical analyses were undertaken using SPSS and MS Excel 2010.

### Total nitrogen evaluation in the flag leaves of transgenic lines

The flag leaves of transgenic lines and the control plants were harvested and the dry weight was determined after oven-drying to a constant weight at 70 °C. A total of 0.1 g of the sample was used to determine the total nitrogen content by the micro-Kjeldhal method [21]. Statistical analyses were undertaken using SPSS and MS Excel 2010.

## Results

### Identification of transgenic plants and *PEPC* gene expression in rice

**Fig. 1:**
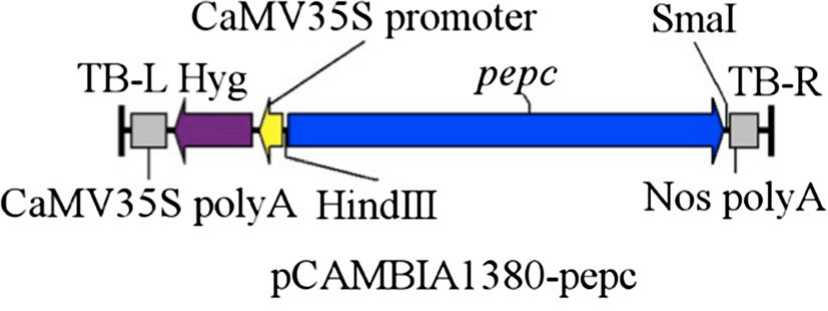
Schematic diagram of the intact *PEPC* gene from sugarcane, the *PEPC* gene from sugarcane is an 8.084 kb fragment containing 10 exons, 9 introns, and the promoter (from −1,300) and terminator (2.5 kb) sequences. *Black rectangles* exons; *lines* introns and the selective antibiotic resistant gene hygromycin phosphotransferase (hph) used for rice transformation

**Fig. 2:**
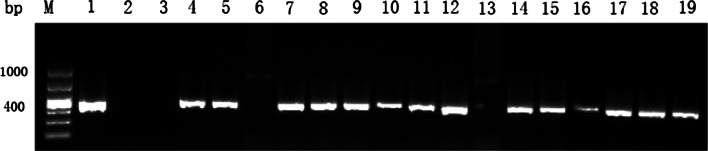
PCR analysis for putative transgenic plants with *PEPC* gene of sugarcane; *M* 1,000 bp DNA marker; *1* blank control (H_2_O); *2* negative control (untransformed plant); *3* positive control (Plasmid); *4*–*19* putative transgenic plants (*6* and *13* were PCR negative)

**Fig. 3:**
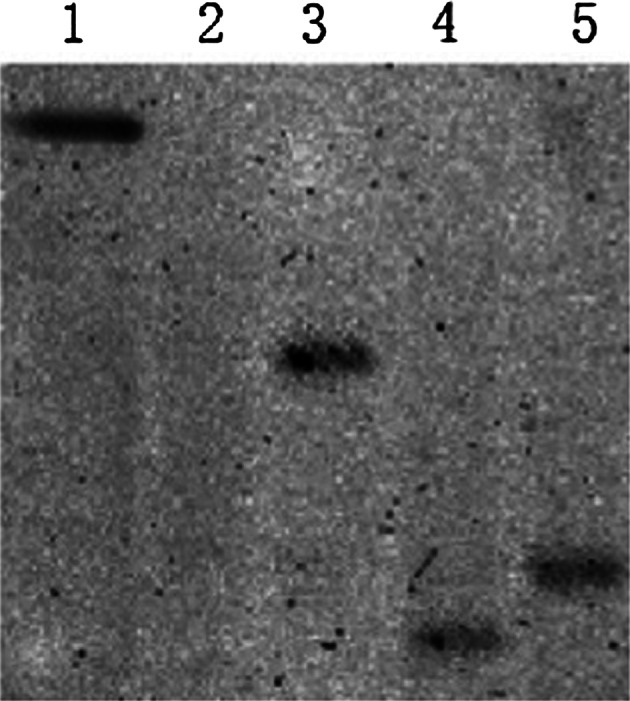
Southern blot analysis of PCR positive transgenic plants with *hph* gene probe; *1* positive control (plasmid); *2* negative control (untransformed plant); *3*–*5* transgenic plants

**Fig. 4:**
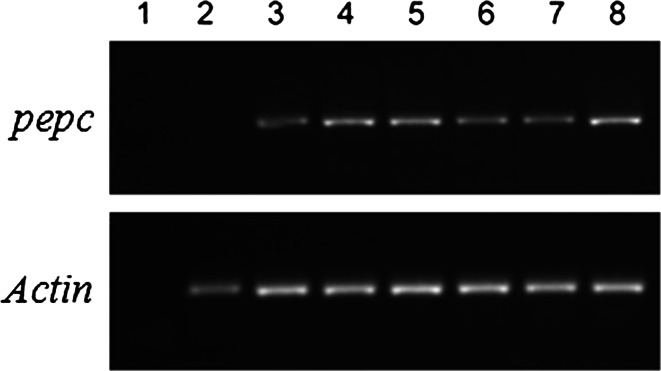
RT-PCR analysis of transgenic plants with sugarcane *PEPC* gene and *actin* (control); *1* blank control; *2* untransformed plant; *3*–*8* transgenic plants

The sugarcane *PEPC* gene construct (Fig. 1) was introduced into the indica restorer line, Hang2. A total of 600 putative hygromycin-resistant transgenic plants (T0) were obtained. The PCR results indicated that there was a 384 bp amplicon for 132 putative transgenic plants (Fig. 2). In order to analyze copy number and integration patterns in the PCR positive transgenic plants, genomic DNA (gDNA) was digested with the *Hin*dIII enzyme and hybridized with the *Hph*-specific probe. Plants with a single copy of the *PEPC* gene were used for further research (Fig. 3). Expression analysis was carried out using RT-PCR (Fig. 4) in order to verify expression of the sugarcane *PEPC* gene in the transgenic lines. The results indicated that sugarcane *PEPC* was expressed at the mRNA level in transgenic plants, but not in wild-type plants. These transgenic plants exhibited normal phenotypes with a normal life cycle, grew to maturity, flowered and set seed. Transgenic plants were grown up to the T4 generation for further research.

**Fig. 5:**
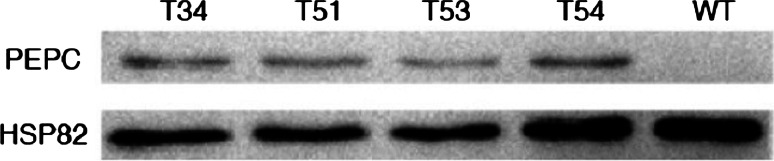
Western blotting analysis of PEPC protein expression of transgenic lines with sugarcane *PEPC* gene. T34, T51, T53, T54 were transgenic lines; WT was untransformed plant. HSP82 was shown as loading control

Western blotting was performed on four selected transgenic lines, named: T34, T51, T53 and T54, using non-transgenic rice plants as controls. The internal control gene, HSP82, was found to be expressed in both the transgenic lines and the control plants. Strong hybridization signals for PEPC were observed in the transgenic lines, but not in the non-transgenic rice (Fig. 5). This suggested that PEPC protein expression levels in transgenic lines with the sugarcane *PEPC* gene were higher than in the non-transgenic rice.

### Analysis of *PEPC* gene expression in different tissues of the transgenic lines

**Fig. 6:**
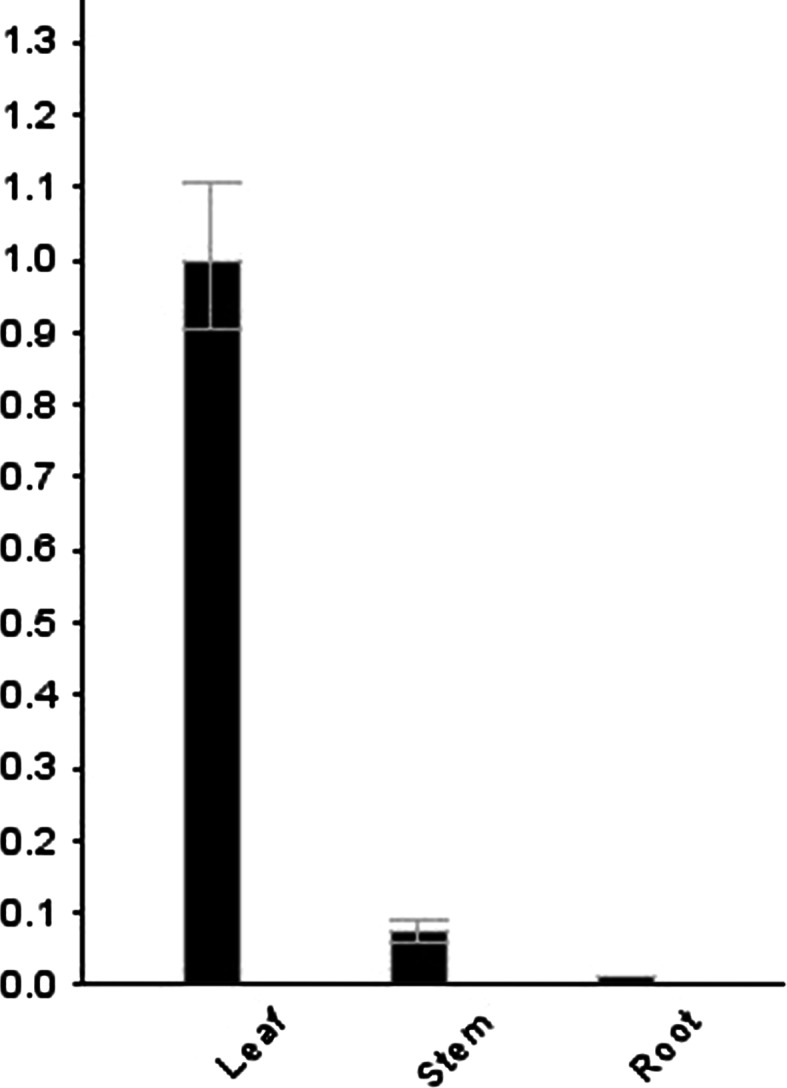
qRT-PCR analysis of *PEPC* gene expression in different tissues of transgenic line T34

The quantitative reverse transcription PCR (qRT-PCR) data for the transgenic line, T34, indicated that *PEPC* gene expression occurred mostly in the leaves (about ten times higher than that in stems) of the transgenic lines. Expression of the *PEPC* gene was the weakest in the roots of the transgenic lines (Fig. 6), indicating that *PEPC* gene expression is found mostly in the green tissues of transgenic lines and demonstrating that PEPC is an enzyme that is involved in photosynthesis.

### PEPC enzyme activity analysis in transgenic lines

**Fig. 7:**
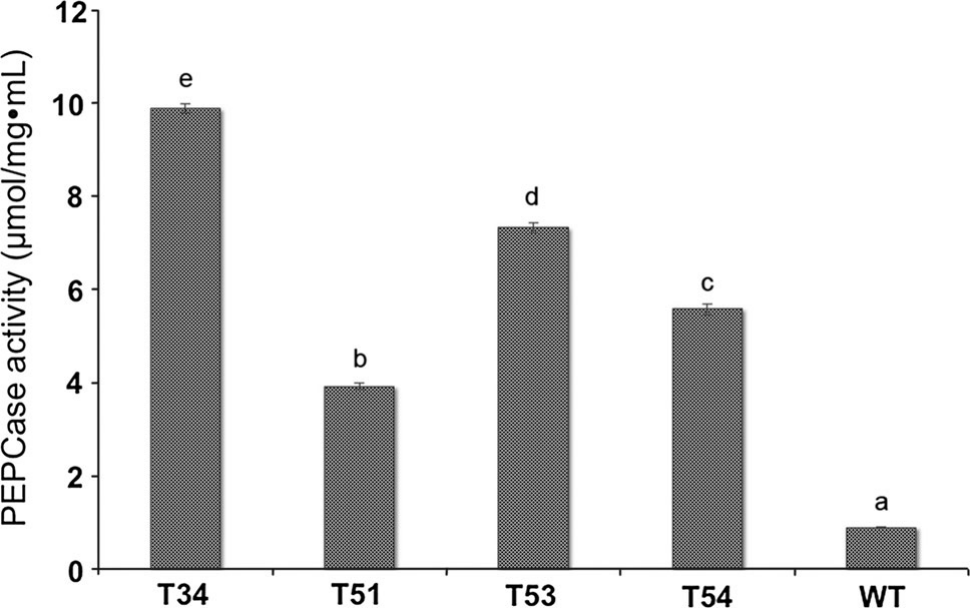
Analysis of PEPC enzyme activities. T34, T51, T53, T54 were transgenic lines; WT was untransformed plant

PEPC enzyme activities in the transgenic lines: T34, T51, T53 and T54, were higher than in the non-transformed rice (Fig. 7) at the 5 % significance level. The highest PEPC enzyme activity was observed in the transgenic line, T34, which was 11.1 times higher than in the non-transgenic plants. This was consistent with the higher PEPC protein levels found in the transgenic lines.

### Analysis of *PEPC* gene expression under stress treatment in transgenic rice

**Fig. 8:**
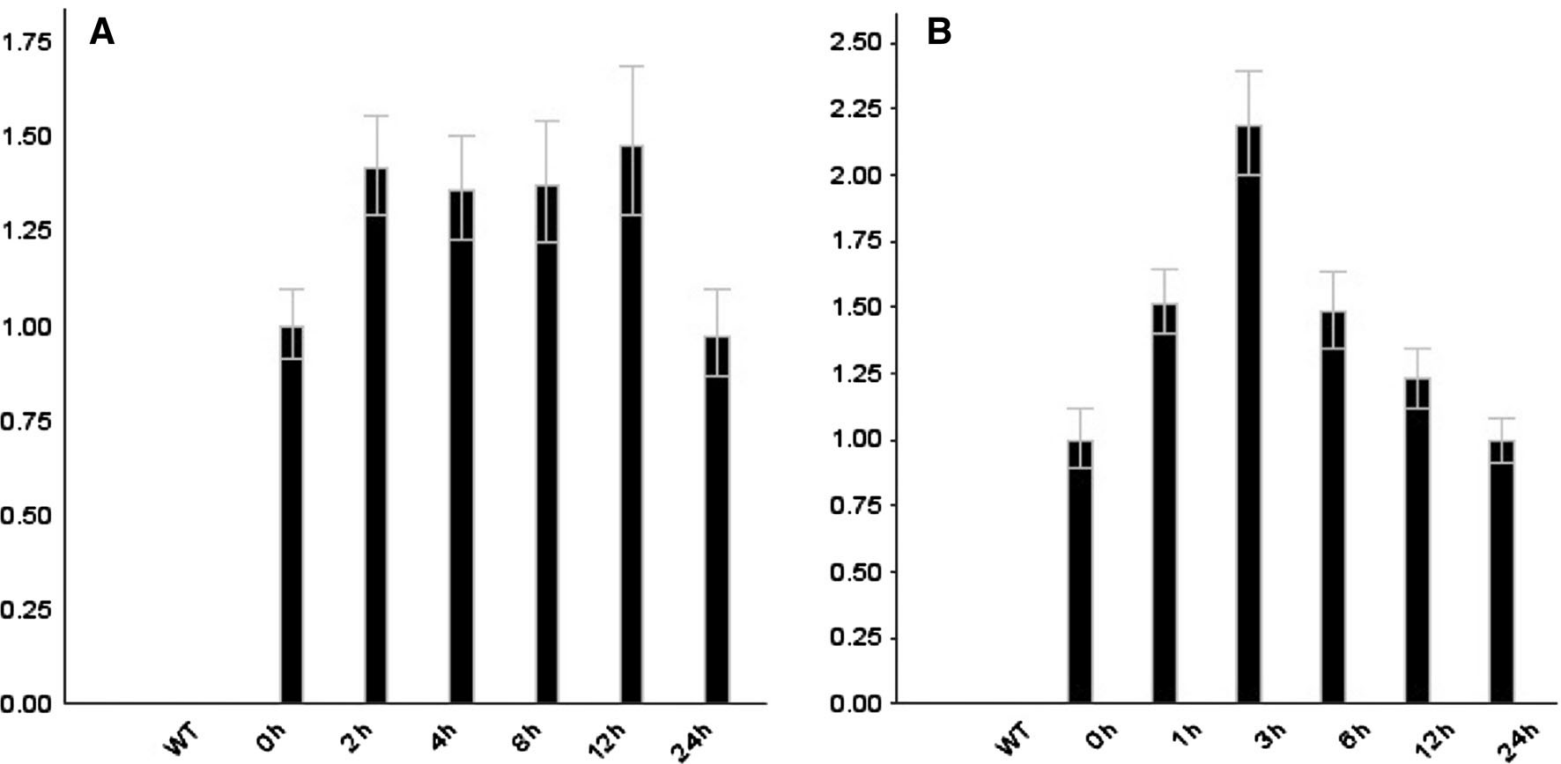
qRT-PCR analysis of *PEPC* gene expression under drought treatment and under high-temperature. WT was untransformed plant as control; transgenic line T34 was treated by drought for 0, 2, 4, 8, 12, 24 h (**a**) and treated at 42 °C for 0, 1, 3, 6, 12, 24 h (**b**)

Under abiotic stress, expression of many proteins related to oxidative stress, photosynthesis, carbohydrate metabolism and regulation are altered [22–25]. In order to examine how expression of *PEPC* was affected by abiotic stress, the T34 transgenic line was subjected to drought and high temperature stress. The qRT-PCR analyses indicated that expression of the sugarcane *PEPC* gene peaked under drought stress after 12 h and then declined (Fig. 8a), reaching levels equivalent to that seen at 0 h after 24 h under drought stress.

In addition, the transgenic line, T34, was kept at 42 °C for 0, 1, 3, 6, 12 and 24 h. The qRT-PCR data indicated that *PEPC* gene expression increased significantly under high temperatures, peaked after 3 h and then declined (Fig. 8b). These results indicated that sugarcane *PEPC* gene expression in transgenic rice was sensitive to drought stress and high temperatures.

### Photosynthetic rate in the transgenic lines

An important consideration for plant breeders is the photosynthetic efficiency of both conventional and transgenic plant varieties [26]. Therefore, the photosynthetic rates of the transgenic plants grown in the transgenic network house were measured at the heading stage. The results showed that the photosynthetic rates of transgenic lines were higher than that of the control plants (Table 2). Moreover, the photosynthetic rates of the transgenic lines: T34, T53 and T54 increased significantly [*p* = 0.00, 0.00 and 0.00, respectively (*p* < 0.05), compared with the control], but the photosynthetic rate of the transgenic line, T51, did not increase significantly [*p* = 0.15, (*p* > 0.05) compared with the control].

**Table 2:** Photosynthetic parameters of the sugarcane pepc gene transgenic lines

Line	Pn (photosynthesis rate) (μmol m^−2^ s^−1^)	SC (stomatal conductance) (cm^−1^)	Ci (internal CO_2_ concentration) (μmol mol^−1^)	Chlorophyll relative content (SPAD)
T34	19.3 ± 0.2b	0.074 ± 0.004	355.84 ± 0.02	31.87 ± 0.92
T51	18.3 ± 0.1ab	0.084 ± 0.001	355.8 ± 2.458	33.97 ± 1.07
T53	21.3 ± 0.1c	0.075 ± 0.001	357.85 ± 0.01	31.10 ± 0.35
T54	22.3 ± 0.3cd	0.090 ± 0.010	355.15 ± 0.03	33.67 ± 0.45
WT	17.9 ± 0.1a	0.061 ± 0.005	356.56 ± 0.02	32.60 ± 0.95

Measurement of agronomic data of all the control and transgenic line were recorded based on three replica experiments in identical conditions and the seeds from selected line were randomly chosen. The data was calculated by SPSS, Excel 2010 software package

It has previously been reported that the chlorophyll content of rice decreased slightly when the maize *PPDK* gene was introduced [27]. The relative chlorophyll content of the sugarcane *PEPC* gene was measured in the transgenic lines: T34, T51, T53 and T54 using a SPAD-502 chlorophyll meter. The results indicated that the relative chlorophyll content of these transgenic lines did not change significantly compared to non-transformed rice (Table 2).

### Total nitrogen in the flag leaves of the transgenic lines

**Fig. 9:**
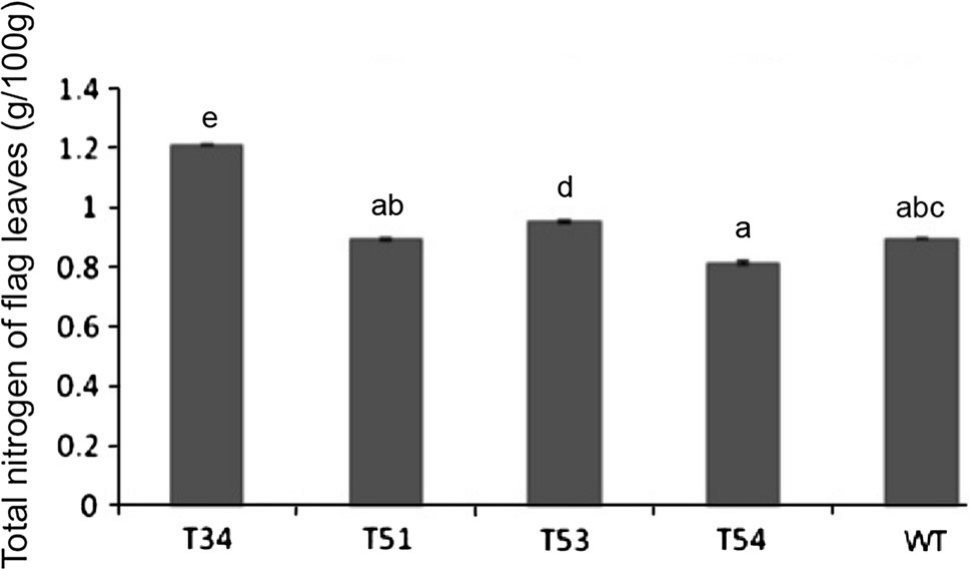
Total nitrogen contains of flag leaves. T34, T51, T53, T54 were transgenic lines; WT was untransformed plant

Since nitrogen is a limiting factor on the photosynthetic capacity of plants, the total nitrogen content of the flag leaves was measured. The transgenic lines: T34 and T53, had significantly higher nitrogen contents compared to the control (*p* < 0.05). However, the total nitrogen contents of the transgenic lines: T51 and T54, were not statistically different from that of non-transgenic rice (Fig. 9).

### Yield characteristics of transgenic rice

**Fig. 10:**
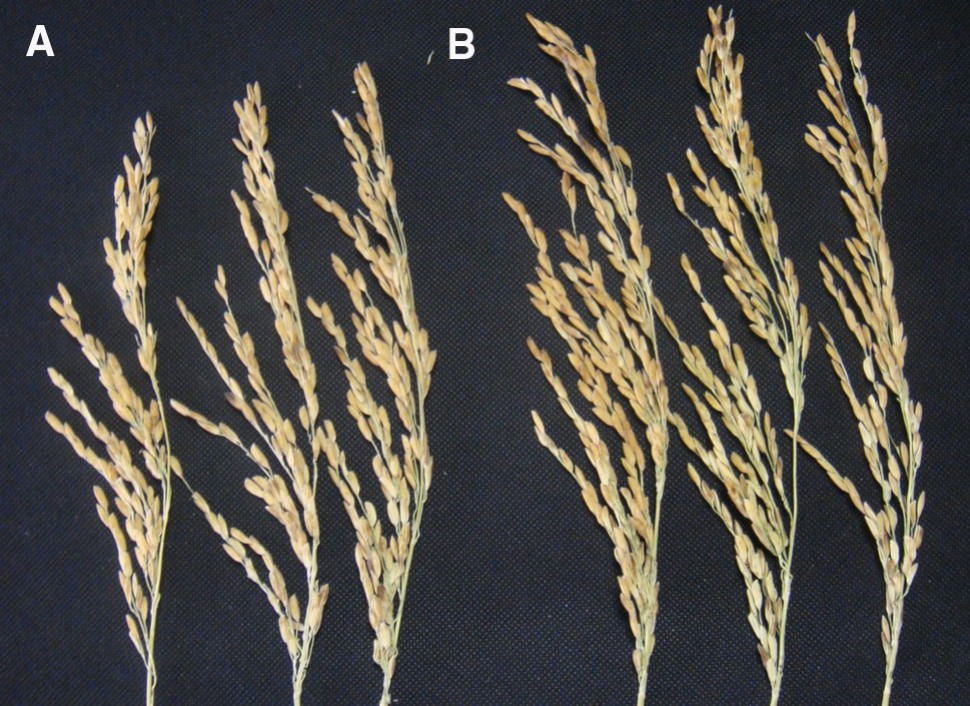
The phenotype of spike between transgenic line and untransformed plant. **a** The phenotype of spike untransformed plant; **b** The phenotype of spike for transgenic line

Three mature plants of each transgenic line were harvested in the transgenic network house. The yield characteristics of each transgenic line were observed after drying in a 65 °C oven. With the exception of T54, the biological weights of T34, T51 and T53 increased significantly (*p* = 0.008, 0.004 and 0.01, respectively) at the 5 % significance level. The panicle lengths of the T51, T53 and T54 lines showed an insignificant trend towards being longer than that of non-transgenic plants (control), while that of the T34 line was significantly longer (*p* < 0.05). The filled grain and total grain numbers of T34, T51, T53 and T54 were all more than those of non-transgenic plants, but the grain filling (%) and 1,000-grain weights of all the transgenic lines were comparable to those of the non-transgenic plants. In addition, the harvest index increased in some but not all transgenic lines (Table 3). Furthermore, to acquire more detailed description of morphological characteristics in transgenic lines, The T5 generation of transgenic line T34, T51, T53 and T54 were planted in the transgenic network house, and ten mature plants of each transgenic line were harvested to analyze yield characteristics further. The results demonstrated that spike numbers of all transgenic lines were more than that of non-transgenic plants (WT). Meanwhile, total filled grain and average filled grain per spike of transgenic lines were also more than that of non-transgenic plants. The panicle length of T34, T51, T53 and T54 were longer than that of non-transgenic plants, but the panicle length of transgenic line T34, T53 was significantly longer than that of non-transgenic plants (*p* = 0.044 and 0.025, respectively) (Supplementary Table 4; Fig. 10). According to the data, not all the yield characteristic indexes for the transgenic lines increased significantly, but filled grain and total grain numbers for all transgenic lines did increase significantly. This explains why introducing sugarcane *PEPC* into rice (C3) results in higher PEPC protein levels and leads to changes in some yield characteristics. It also further illustrates that PEPC plays an important role in rice.

**Table 3:** Yield characteristics of transgenic lines containing the sugarcane pepc gene

Line	Biological weight (g)	Panicle length (cm)	Filled grain	Total grain	Grain filling (%)	1,000-grain weight (g)	Harvest index (%)
T34	128.1 ± 2.1bc	25.1 ± 1.1	1,250.7 ± 148.0b	2,547.3 ± 231.6b	47.9 ± 4.7	24.7 ± 0.3	24.1 ± 2.4a
T51	135.8 ± 27.6bcd	23.6 ± 1.2	2,496.3 ± 201.8e	4,261.0 ± 964.9e	60.2 ± 11.0	25.7 ± 0.3	48.7 ± 12.1cd
T53	125.8 ± 28.9b	22.1 ± 1.9	2,289.0 ± 721.1d	3,997.3 ± 898.9d	56.5 ± 4.8	23.5 ± 1.6	42.6 ± 7.8c
T54	104.2 ± 30.7a	23.7 ± 3.6	2,121.3 ± 2,071.3c	3,892.0 ± 2,471.1c	47.3 ± 17.5	22.7 ± 0.2	41.8 ± 32.2b
WT	67.3 ± 4.1a	21.0 ± 1.2	565.0 ± 163.4a	1,166.0 ± 182.0a	47.8 ± 6.3	26.2 ± 0.5	21.9 ± 5.3a

Grain filling (%) = filled grain/total grain × 100 %, Harvest index (%) = filled grain weight/biological weight × 100 %. Values shown are averages of three replicates. Statistical analyses were undertaken using SPSS and MS Excel 2010. a, b, c, d, e indicated significant difference

## Discussion

In order to increase the yield potential of rice, researchers have tried various methods to improve the photosynthetic ability per leaf area, but with limited success [26, 28–32]. Compared to non-transformed, wild-type rice, transgenic rice expressing the maize *PEPC* gene required a higher light intensity to bring about photosynthesis saturation, had a higher photosynthetic CO_2_ uptake rate and a higher carboxylation efficiency [33]. The PEPC substrate level (phosphoenolpyruvate) was slightly lower and the reaction product (oxaloacetate) was slightly higher in the transgenic rice, which suggested that the maize *PEPC* gene was functional in the transgenic rice lines [34]. *PEPC* from C_4_ millet (*Seteria italica*) was introduced into Japonica rice and the transformants only showed improvements in both photosynthesis rates and yields under upland field cultivation conditions [35]. In this study, the photosynthetic rates of Hang2 rice lines were significantly improved by introducing the intact sugarcane *PEPC* gene.

PEPC enzyme activity has been found to be high in several transgenic plants. It has been reported that a transgenic rice line with the maize *PEPC* gene expressed large amounts of the maize PEPC protein and exhibits high PEPC activity [36]. PEPC activity was significantly higher in *PEPC* transgenic rice than in the wild type rice [37, 38]. A 50-fold elevation in PEPC activity, but without a significant effect on overall growth, has been reported in *PEPC* transgenic rice compared to non-transgenic rice [31]. This study also found that PEPC activity was significantly higher in the transgenic rice lines. There was 11.1 times more activity in the transgenic plants than in the non-transgenic plants.

Nitrogen is required for the biosynthesis of proteins, nucleic acids and chlorophyll. It is unquestionably one of the most important nutrients needed for plant growth. Nitrogen starvation causes lower intracellular concentrations of amino acids and proteins, including those involved in photosynthesis and carbon metabolism [39, 40]. Nitrogen is a limiting factor on the photosynthetic capacity of plants. C4 plants have higher efficiencies than C3 plants when it comes to the utilization of light, water and nitrogen, perhaps owing to the low photorespiration rate of the former, which affects carbon and nitrogen metabolism [41]. Introduction of the intact maize *C4*-*PPDK* gene into japonica rice led to significant accumulation of the PPDK protein in the leaf chloroplasts of homozygous lines. Accumulation of PPDK affected the chlorophyll, Rubisco and nitrogen contents in the leaves. The transgenic line, PD25, showed an increase in leaf nitrogen content of approximately 7 %. Rubisco content increased slightly and chlorophyll content decreased slightly on a leaf area basis [27]. In our study, total nitrogen content in most of the transgenic lines was not significantly different to that of the non-transgenic line. Further research is needed to measure the activities of enzymes related to nitrogen metabolism.

Investigation of the main agronomic traits of three types of transgenic rice containing the maize C4 photosynthetic genes: *PEPC*, *PPDK*, or *PEPC*+*PPDK*, indicated that those containing the maize *PEPC* and/or *PPDK* genes had more panicles and higher yields than the untransformed controls [42]. Similarly, *PEPC* and *PPDK* transgenic rice have been reported to produce 22–24 % more grains than wild-type plants [33]. *PEPC* transgenic rice had higher dry weights for leaves, stems, sheaths and panicles than the untransformed wild rice, with the largest increase occurring in the panicles. The soluble sugar and protein contents in the grains of *PEPC* transgenic rice were also significantly enhanced [38]. The filled grain and total grain numbers of sugarcane *PEPC* gene transgenic lines were higher than those of untransformed rice. In addition, the biological weights of the transgenic lines were also higher than that of the untransformed rice. The harvest index of most transgenic plants was also higher than that of control plants, which indicated that the yield of transgenic Hang2 plants with the intact sugarcane *PEPC* gene improved to some degree. Thus, all these studies proved that C4-type genes function in transgenic rice. Recently, the fact that genes in C3 species can be recruited into cell-specific functions in the C4 pathway suggests that the trans-factor(s) responsible for generating cell-specific accumulation of proteins is important when engineering C4 photosynthesis in rice [43]. Further research is needed to reveal the action mechanism of protein encoded by C4-type genes in transgenic rice and what their action pathways are in C3 rice.

## Electronic supplementary material

Below is the link to the electronic supplementary material.

Supplementary material 1 (DOC 34 kb)
