# Supplementary material for: Physiological and photosynthetic characteristics of indica Hang2 expressing the sugarcane PEPC gene
Source: Mol Biol Rep. 2014 Jan 29;41(4):2189–97. doi: 10.1007/s11033-014-3070-4 (PMC3968443; doi:10.1007/s11033-014-3070-4)
Supplement: Supplementary file 1 — Supplementary material 1 (DOC 34 kb) [file 11033_2014_3070_MOESM1_ESM.doc]

Table 4 Yield characteristics of transgenic lines containing the sugarcane *PEPC* gene

| Line | Spike number | Panicle length（cm） | Total filled grain | Total grain | Average grain per spike | Average filled grain per spike | 1000-grain weight（g） | Grain weight per plant（g） | Grain filling（%） | Number of primary branch |
| --- | --- | --- | --- | --- | --- | --- | --- | --- | --- | --- |
| WT | 6.7±0.9 | 22.9±1.4a | 879.0±168.9a | 1261.7±236.5 | 186.0±12.5 | 129.3±9.2 | 27.8±1.0 | 27.0±5.0 | 69.6±0.5a | 11.0±1.2 |
| T34 | 7.5±0.1 | 25.1±0.2bcd | 1002.4±4.2ab | 1429.9±35.5 | 190.3±1.2 | 133.4±0.8 | 27.0±0.4 | 30.0±0.3 | 69.7±1.2a | 9.4±0.9 |
| T51 | 6.9±0.7 | 24.7±0.2ab | 984.3±38.3ab | 1246.1±76.2 | 186.6±11.5 | 148.7±14.0 | 27.0±0.1 | 28.2±1.4 | 79.4±2.4bc | 10.0±0.3 |
| T53 | 8.6±0.6 | 25.4±0.4bcde | 1164.8±93.7ab | 1627.6±90.2 | 191.8±7.7 | 136.3±1.3 | 28.3±1.1 | 35.9±3.1 | 71.4±2.9a | 10.4±0.1 |
| T54 | 8.2±1.0 | 24.9±0.2abc | 1212.1±94.3b | 1540.5±114.9 | 191.8±12.4 | 152.2±11.2 | 27.5±0.7 | 35.4±3.0 | 78.8±1.1c | 10.6±0.1 |

Note: Grain filling (%) = Filled grain / Total grain × 100%; Statistical analyses were undertaken using SPSS and MS Excel 2010. a,b,c indicated significant difference
